# Squamous cell carcinoma in chronic lesions of recalcitrant chromoblastomycosis. Study of ten patients treated at the Dermatological Center of Yucatán, Mexico^؆^

**DOI:** 10.1016/j.abd.2026.501372

**Published:** 2026-06-03

**Authors:** Eduardo Rafael Calderón Quiroz, Edoardo Torres Guerrero, Héctor Proy Trujillo, María Elisa Vega Memije, Carlos Atoche Diéguez

**Affiliations:** a Department of Dermatology, Dr. Valentín Gómez Farías Hospital, University of Guadalajara, Guadalajara, Mexico; b Department of Dermatology, Yucatán Dermatology Center, Yucatán, Mexico; c Department of Dermatologic Surgery, Yucatán Dermatology Center, Yucatán, Mexico; d Division of Dermatology and Dermatopathology, Dr. Manuel Gea González General Hospital, National Autonomous University of Mexico, Mexico City, Mexico; e Mycology Laboratory, Yucatán Dermatology Center, Yucatán, Mexico

**Keywords:** Carcinogenesis, Carcinoma, squamous cell, Chromoblastomycosis, Chronic disease, Dermatomycoses, Skin neoplasms

## Abstract

**Background:**

Chromoblastomycosis is a chronic granulomatous fungal infection of the skin and subcutaneous tissue caused by dematiaceous fungi of the family *Herpotrichiellaceae*. It is considered an occupational disease, predominantly affecting male agricultural workers, with a worldwide distribution, especially in tropical and subtropical regions. *Fonsecaea pedrosoi* accounts for approximately 90% of reported cases.

**Objective:**

To describe the etiological agents, clinical features, and major complications associated with chromoblastomycosis.

**Methods:**

A retrospective, descriptive, and observational study presenting a case series was conducted, focusing on the pathogenesis, clinical presentation, disease course, and reported complications of chromoblastomycosis.

**Results:**

A total of 131 cases of chromoblastomycosis were documented; 10 patients (7.65%) developed squamous cell carcinoma. The mean age was 65.8-years, and the mean disease duration prior to malignant transformation was 17.4-years. The most frequently affected site was the upper limb (70%), followed by the lower limb (20%) and the trunk (10%).

**Study limitations:**

The descriptive nature of this review limits causal inference and precise estimation of malignant transformation rates.

**Conclusion:**

Chromoblastomycosis is a chronic, polymorphic cutaneous infection often diagnosed late, leading to long-standing disease and disabling sequelae. Malignant transformation to squamous cell carcinoma represents one of its most severe complications and may occur even in residual scars. Early diagnosis and prolonged follow-up are essential to reduce morbidity and prevent malignant progression.

## Introduction

Chromoblastomycosis (CBM), also known as chromomycosis, verrucous dermatitis, Lane-Pedroso’s mycosis, Fonseca’s disease, and Carrión’s mycosis, is a chronic, granulomatous, and occasionally suppurative mycosis of the skin and subcutaneous tissue caused by traumatic inoculation of dematiaceous fungi of the family *Herpotrichiellaceae*.^1^

It is a cosmopolitan and occupational disease that predominates in tropical and subtropical regions, with the highest prevalence between 30° latitude North and 30° latitude South, although several case reports exist from temperate regions. In México, CBM is the third most frequent subcutaneous mycosis.^2^ The disease mainly affects farm workers, mostly males, and often leaves disabling sequelae. Classically, causative agents have been described in soil, plants, and decaying wood, and mainly belong to the genera *Fonsecaea, Phialophora*, and *Cladophialophora.*^2^

*Fonsecaea pedrosoi* (90% of worldwide cases) and *Cladophialophora carrionii* are the most prevalent species in endemic regions; however, other species such as *Fonsecaea monophora*, *Fonsecaea nubica*, *Phialophora verrucosa*, *Fonsecaea pugnacius*, *Rhinocladiella aquaspersa*, *Cladophialophora samoensis*, *Cyphellophora ludoviensis, Rhinocladiella tropicalis, Rhinocladiella similis, Exophiala jeanselmei and Exophiala spinifera* have been reported.^1^, ^2^

This condition typically presents as insidious-onset cutaneous lesions that eventually progress to physical disability, and the clinical picture can be polymorphic. Patients may present verrucous, nodular, tumoral, psoriasis-like, scarring lesions, and the so-called “mossy foot”.^1^, ^2^, ^3^ Malnutrition, immunosuppression, and genetic association with HLA-A29 are considered probable risk factors.^4^

Direct microscopy with Potassium Hydroxide (KOH) 10%–20%, Sabouraud agar culture, histopathology of skin biposy, and molecular tests are useful diagnostic tools to confirm clinical suspicion; however, diagnosis is often delayed, allowing the development of complications, among which Squamous Cell Carcinoma (SCC) is the most severe. This neoplasm may even arise on apparently scarred lesions.^2^, ^3^, ^5^

## Methods

A retrospective, descriptive, and observational study presenting a case series was conducted at the “Dr. Fernando Latapí” Dermatology Center of Yucatán in Mérida, México. Cases registered between 2001 and 2025 were identified from the institutional oncologic follow-up registry; 10 patients with a history of chromoblastomycosis lesions and subsequent development of squamous cell carcinoma were reported.

**Figure 1:**
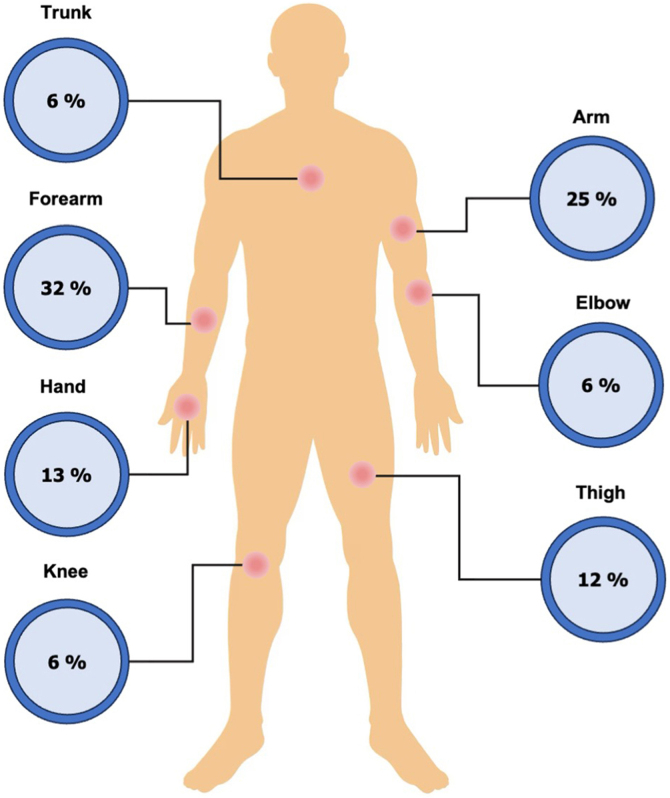
Percentages of Chromoblastomycosis (CBM) lesion presentation. Topographic distribution of CBM lesions.

The 10 patients were all male and farmers, aged 58 to 86-years, with a mean age of 65.8-years. Diagnosis of CBM was made through physical examination and mycological studies, all with prolonged evolution of lesions ranging from 5- to 30-years, with a mean of 17.4-years (Table 1). Regarding topography, upper extremities predominated with 70%, lower extremities accounted for 20%, and trunk for 10% (Fig. 1).

**Table 1:** Clinical and demographic characteristics of the patients with squamous cell carcinoma in chronic lesions of chromblastomycosis.

Patient	Age (years)	Sex	Disease duration (years)	Site affected	Mycological study	Culture
1	65	M	26	Right thigh and leg	Muriform cells	*F. pedrosoi*
2	59	M	10	Right elbow	Muriform cells	*F. pedrosoi*
3	86	M	25	Right hand and wrist	Muriform cells	*F. pedrosoi*
4	66	M	5	Right forearm and hand	Muriform cells, filaments	*F. pedrosoi*
5	60	M	20	Left arm and forearm	Muriform cells, filaments	*F. pedrosoi*
6	70	M	11	Left arm and forearm	Muriform cells, filaments	*Negative*
7^a^	58	M	12	Left arm and forearm	Muriform cells	*F. pedrosoi*
8	66	M	30	Left thigh, knee, and leg	Muriform cells, filaments	Negative
9	63	M	20	Right arm and forearm	Muriform cells	*Fonsecaea spp*
10	65	M	15	Trunk	Muriform cells	Negative

a Patient with development of lymph node metastasis.

**Figure 2:**
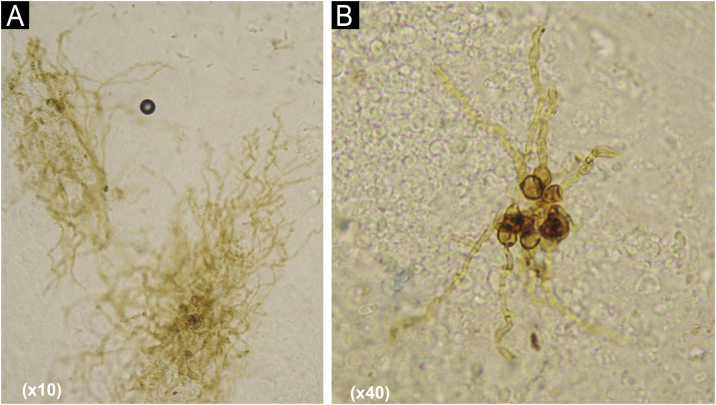
Microscopic characteristics of the direct KOH examination. All patients underwent direct examination with chlorazol black or 20% KOH, revealing muriform cells; in up to 56% of cases these were accompanied by septate hyphae.

**Figure 3:**
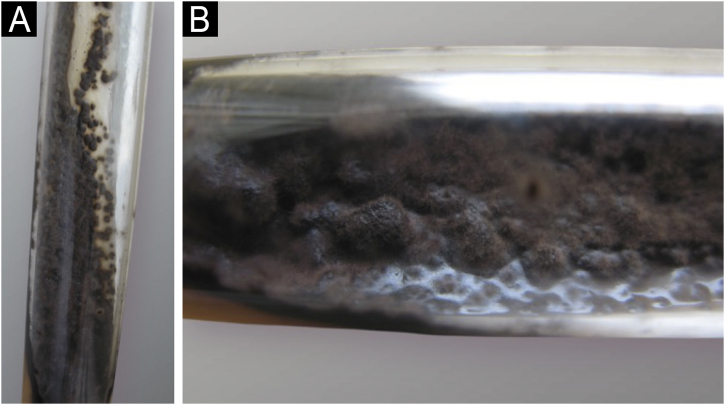
Macroscopic characteristics of the culture. The isolates grown on Sabouraud agar showed black or grayish velvety colonies, in which the reproductive forms of *Fonsecaea sp*., *Cladosporium*, *Rhinocladiella*, and occasionally phialides ‒ were identified.

Most patients presented the erythematous-scaly and verrucous varieties. Direct KOH examination revealed fumagoid (muriform) cells in all cases (Fig. 2). Culture showed development of 6 colonies with morphological characteristics compatible with *F. pedrosoi*, one identified as *Fonsecaea* sp., two producing only sterile mycelium (non-identifiable morphologically), and no growth in the remaining samples (Table 1 and Fig. 3). All patients received oral itraconazole therapy, with clinical improvement and reduction of lesions, and in some cases complete resolution.

**Figure 4:**
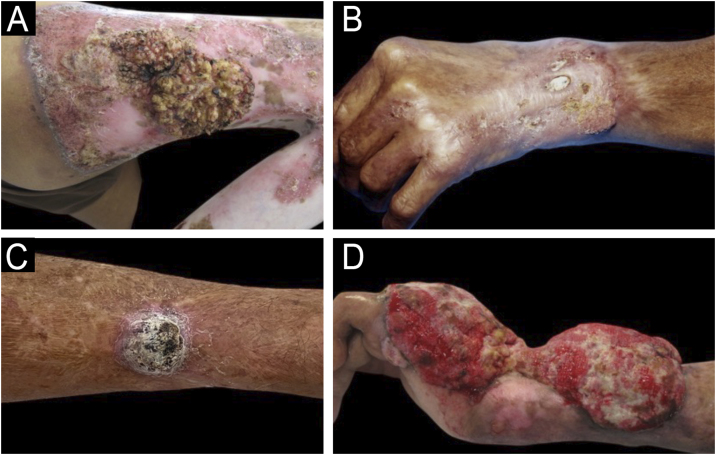
Clinical characteristics of tumoral neoformations arising on CBM scars. (A) Exophytic, vegetating tumoral lesion with areas of ulceration and bleeding over the scar. (B) Exophytic, keratotic lesion with ulcerated areas; note the exposure of ligaments. (C) Keratotic nodular lesion on an erythematous base. (D) Elevated plaque-like neoformation with denuded and bleeding areas, painful, over the scar, all confirmed as squamous cell carcinoma.

**Figure 5:**
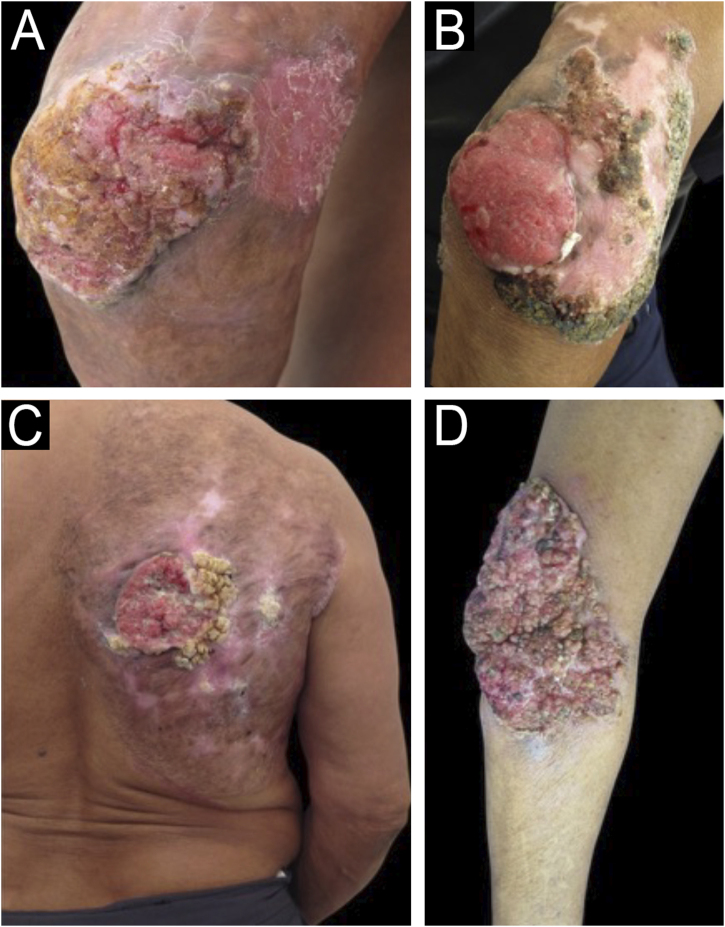
Clinical characteristics of tumoral neoformations on CBM scars. (A) Verrucous, erythematous, keratotic lesion arising on an erythematous scar base. (B) Erythematous, ulcerated nodular lesion on an erythematous-keratotic base. (C) Exophytic, vegetating, keratotic tumoral lesion with some ulcerated and bleeding areas. (D) Vegetating lesion, confirmed as squamous cell carcinoma.

**Figure 6:**
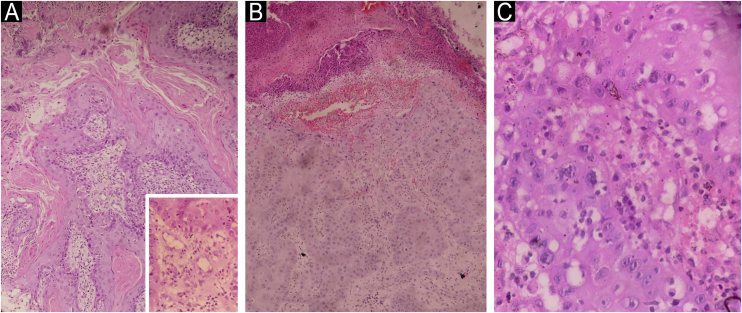
Histopathological characteristics of tumor lesions. Skin biopsies stained with Hematoxylin & eosin: (A) (Hematoxylin & eosin, 10×) Exulcerated epithelium with compact laminar stratum corneum and cellular debris; infiltrative sheets and cords of atypical keratinocytes forming keratin pearls, with hyperchromatic nuclei, mitoses, and lymphocytic infiltrate. (Hematoxylin & eosin, 50×) Dilated capillaries with erythrocyte extravasation. Findings consistent with well-differentiated, ulcerated, infiltrative squamous cell carcinoma. (B) (Hematoxylin & eosin, 10×) Ulcerated epithelium with serohematic crust, debris, and bacterial colonies, anastomosing cords of pleomorphic, hyperchromatic squamous cells with atypical mitoses and eosinophilic cytoplasm, lymphocytic infiltrate, and hemorrhage. Compatible with ulcerated, infiltrative squamous cell carcinoma. (C) (Hematoxylin & eosin, 50×) Atypical squamoid proliferation with pleomorphism and abnormal mitoses; papillary dermis showing erythrocyte extravasation and hemosiderin. Consistent with infiltrative squamous cell carcinoma.

Despite adherence to treatment, some patients developed malignant transformation to squamous cell carcinoma during follow-up (mean 5-years), while others showed transformation after completion of antifungal therapy (up to 13-years after treatment onset). Tumoral, keratotic, vegetating, or ulcerative lesions developed over pre-existing plaques (Figure 4, Figure 5). Histopathological examination confirmed neoplastic development in all cases, consistently showing anastomosing mantles and cords composed of atypical and pleomorphic keratinocytes with large, hyperchromatic nuclei (Fig. 6).

**Figure 7:**
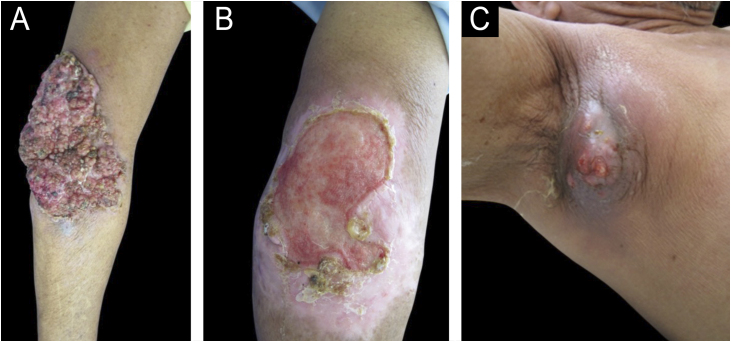
Lymph node squamous cell carcinoma metastasis to the axillary region. (A) Vegetating, ulcerated, friable, and bleeding squamous cell carcinoma lesion, extremely painful at the elbow. (B) Residual ulcerated lesion with granulation tissue following radiotherapy. (C) Metastatic nodular lesion, ulcerated with serohematic discharge in the axillary hollow, observed 2-months after completion of radiotherapy.

All patients were referred to medical oncology, where radiotherapy was administered. Nonetheless, one patient developed distant nodal metastasis 2-months after radiotherapy (Fig. 7), although none of the patients died throughout the study follow-up period.

## Results

During the study period, a total of 131 cases of chromoblastomycosis were documented. Among these, 10 patients (7.65%) subsequently developed squamous cell carcinoma as a malignant transformation of their chronic lesions.

The mean age at the time of carcinoma diagnosis was 65.8-years. The average duration of chromoblastomycosis prior to malignant transformation was 17.4-years, reflecting the long-standing and persistent nature of the infection.

Regarding anatomical distribution, the upper limb was the most frequently affected site, accounting for 70% of cases, followed by the lower limb (20%) and the trunk (10%).

## Discussion

Chronic CBM lesions have been associated with multiple complications, most frequently bacterial superinfection, chronic ulcers, lymphedema, and impaired wound healing; however, patients may develop ulcers, lymphatic alterations, and even malignant lesions such as SCC or melanoma.^6^

Since the first reported case of CBM-associated SCC, additional series have been published worldwide. In México, there is no reliable registry of malignant transformation cases; however, a compilation of cases published over 70-years from at least eight dermatological centers (1943–2013) exists.^6^

In México a multicenter series published in 2014 reported 603 cases of CBM collected over seven decades, positioning the country among those with the highest incidence worldwide, males represented 63%, the most affected age group being the fourth and fifth decades (up to 26.2%). Geographically, Sinaloa had the highest number of cases (33.6%), followed by Yucatán (9.7%), then Veracruz and Jalisco. Farm work was the most commonly associated occupation. Lesion topography corresponded to international literature, with lower extremities affected in 54.7%, followed by upper extremities in 34.1%, with evolution time ranging from 1- to 5-years. Nonetheless, despite efforts in surveillance and registry, it remains a neglected and underestimated disease.^6^

SCC arises from spinous layer keratinocytes in the epidermis and is the second most frequent malignant skin neoplasm worldwide, associated mainly with sun exposure, hydrocarbons, human papillomavirus, burns, and chronic wounds.^7^

Its clinical manifestations vary, showing superficial, verrucous, nodular, or ulcerated forms. The verrucous form is more common in chronic lesions, which can develop into large lesions similar to Buschke-Löwenstein tumor, Ackerman tumor, and cuniculatum epithelioma.^7^

Association between CBM and SCC has been described historically, with 22 cases currently reported^8^ (summarized in Table 2).^4^, ^9^, ^10^, ^11^, ^12^, ^13^, ^14^, ^15^, ^16^, ^17^

**Table 2:** Reported cases of malignant transformation in chromoblastomycosis.^4^, ^9^, ^10^, ^11^, ^12^, ^13^, ^14^, ^15^, ^16^, ^17^

Authors and references	Disease duration (years)	Geography	Agent	Neoplasia Type
Torres et al.^4^	31	Mexico	*F. pedrosoi*	SCC
Caplan^9^	11	Nicaragua	*F. pedrosoi*	Epidermoid anaplastic carcinoma
Foster & Harris^10^	>10	Solomon Islands	Not reported	SCC
Foster & Harris^10^	20	Australia	Not reported	SCC
Paul et al.^11^	28	French Guiana	*F. pedrosoi*	SCC
Queiroz-Telles et al.^12^	36	Brazil	.. .	SCC
Takase et al.^13^	8	Japan	*F. pedrosoi*	SCC
Gon & Minelli^14^	30	Brazil	*F. pedrosoi*	Acral lentiginous melanoma
Esterre et al.^15^	6	Madagascar	*C. carrionii*	SCC
Esterre et al.^15^	5	Madagascar	*C. carrionii*	SCC
Jamil et al.^16^	21	Malaysia	*F. pedrosoi*	SCC
Rojas et al.^17^	18	Venezuela	*C. carrionii*	SCC

*F. pedrosoi*, *Fonsecaea pedrosoi*; *C. carrionii*, *Cladophialophora carrionii*; SCC, Squamous Cell Carcinoma.

In 1968, Caplan reported the first case of anaplastic SCC in a farmer who developed anaplastic SCC in areas of chronic hyperplasia following CBM lesions, treated with antifungals and skin grafting.^9^

In 1978, Foster and Harris reported two cases in Oceanian patients, suggesting malignancy risk based on extensive SCC over chronic CBM lesions of up to 20-years’ duration.^10^

In 1991, Paul et al. reported malignant transformation in cutaneous-synovial tissue in a 67-year-old farmer from French Guiana. Initial lesions were distinct from the site of malignant transformation. After 28-years, extensive verrucous plaques compatible with SCC developed at the initial site, requiring surgical removal with disarticulation. Later that year, new articular lesions arose at a different site, with tendon involvement, and mycological studies again confirmed *F. pedrosoi* infection, responding to itraconazole.^11^

In 2010, a case of SCC developing in a gluteal chromoblastomycosis lesion was reported in México. After initial treatment with cryosurgery, potassium iodide, and itraconazole with partial improvement, the patient discontinued follow-up and was later found severely ill with multi-organ failure, likely secondary to infiltrating SCC due to neglected CBM, ultimately dying during hospitalization.^4^

Although the association between chronic ulcers and malignant transformation is well recognized ‒ as in Marjolin’s ulcer ‒ SCC may develop on burn scars, traumatic wounds, varicose ulcers, non-healing lupus vulgaris lesions, tropical ulcers, osteomyelitis-related cutaneous lesions, and chronic CBM lesions.^8^ However, CBM-associated SCC remains rare, with few reported cases, generally associated with long evolution, though reports continue to appear.^18^

Regarding pathophysiology, the mechanism of malignant transformation remains unclear. Proposed mechanisms include the release of enzymes and free radicals from immune cells, leading to malignant change, and associations with verrucous clinical variants or long-standing disease.^18^

The chronic inflammatory process occurring in chromoblastomycosis lesions may contribute to their malignant transformation through the following mechanisms: When specifically studying *F. pedrosoi*, the importance of IL-17 was demonstrated in contributing to the clearance of extracellular pathogens such as hyphae through neutrophil recruitment and its relationship with M2-type macrophages. Although this system could be effective for eradicating the etiologic agent, an imbalance between Th17 lymphocytes and the action of regulatory T-lymphocytes has been reported, with the former predominating. This leads to an excessive and persistent, yet ineffective, response that favors chronicity.^19^

Inflammatory cells, including polymorphonuclear cells and macrophages, release enzymes, Reactive Oxygen Species (ROS), growth factors, pro-angiogenic molecules, and arachidonic acid-derived metabolites such as prostaglandins and leukotrienes, all of which have the potential to induce cellular damage. Among these, cyclooxygenase-2 stands out; its upregulated expression within the inflammatory cascade may promote cell proliferation, inhibit apoptosis, and favor tumor angiogenesis. As a result of the activity of these secreted substances, in vitro studies have shown that oxidative stress, DNA damage, genomic instability, and epigenetic modifications may occur, ultimately increasing the mutation rate.^20^

This sequence of genetic damage leads to the disruption of DNA repair pathways through the suppression of repair enzymes, while simultaneously increasing the expression of cytidine deaminase. Under normal conditions, its activity is restricted to antibody diversification in B-lymphocytes by promoting genomic instability in that context. However, in chronic inflammatory states, it is aberrantly expressed in epithelial cells, inducing mutations in key oncogenes and tumor suppressor genes, thereby accelerating carcinogenesis.^20^

**Figure 8:**
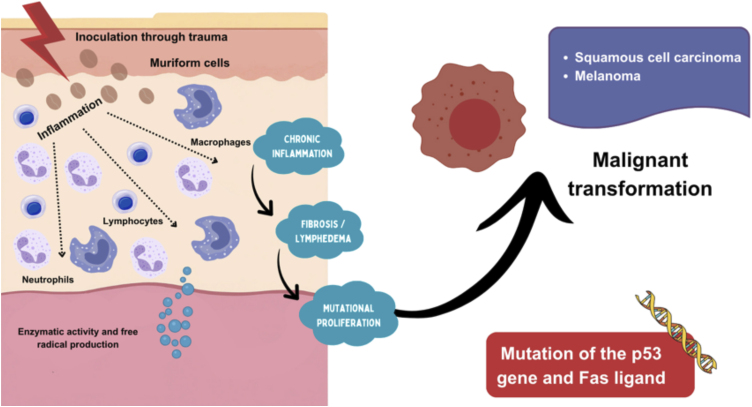
Pathophysiological scheme of malignant transformation. The development of malignant lesions arising in chromoblastomycosis scars involves chronic inflammation driven by neutrophils and macrophages, which release enzymes and free radicals capable of inducing mutagenic damage to the p53 tumor suppressor gene and the Fas ligand, thereby promoting malignant transformation.

Scar tissue itself may act as a tumor promoter, triggering mutations in the Fas gene. Carcinomatous conversion of chronically ulcerated or inflamed epithelium progresses through two phases: initiation and promotion. During initiation, normal cells become latent carcinogenic potentials; promotion involves carcinogenic stimulation via impaired repair and apoptosis^4^, ^7^ (Fig. 8).

Histological examination (with deep biopsies) is essential to confirm clinical suspicion and evaluate the grade of atypia and invasion, which may reach deep structures, as these neoplasms tend to be more aggressive when arising on chronic lesions compared to de *novo* SCC.^4^

To improve diagnostic accuracy, several immunohistochemical markers have been evaluated, including p53 expression. However, its diagnostic utility is limited, as its expression has been documented in both benign and malignant skin lesions.^21^ A previous publication by one of the authors highlights the utility of Syndecan-1 as a histological marker differentiating pseudoepitheliomatous hyperplasia from true SCC. In a study of samples from SCC, CBM, and normal skin, Syndecan-1 was strongly positive in normal tissue, moderately expressed in CBM with pseudoepitheliomatous hyperplasia, and mostly negative in SCC, with only 40% showing weak expression.^22^ In the absence of this tool, findings of cellular atypia, abnormal mitoses, and enlarged hyperchromatic nuclei support neoplastic transformation. Finally, in chronic lesions, a state of sustained tissue hypoxia has been identified, which activates the hypoxia-inducible factor-1 alpha pathway. This factor regulates key genes involved in cell survival, angiogenesis, and immune evasion, establishing an environment conducive to tumor initiation and progression.^21^

Management is difficult, but Mohs micrographic surgery is recommended, achieving cure rates near 98%, as these variants are generally aggressive.^4^, ^7^ Clinical suspicion and histological diagnosis of aggressive malignant tumors are crucial to allow timely treatment and reduce mortality.^4^

Although CBM is not the most common subcutaneous mycosis in México, its frequency is significant, particularly given the frequency of SCC development in patients treated at the Dermatology Center of Yucatán. Therefore, reporting this case series ‒ the largest from a single center worldwide with documented CBM-associated SCC ‒ is imperative.

## Conclusion

CBM remains a relatively frequent but neglected disease in México, with high prevalence in tropical regions and affecting mainly low-income individuals, particularly farmers. It continues to be misdiagnosed at first-contact healthcare services, contributing to chronic evolution.

Delayed management favors complications such as malignant transformation to SCC, still rare but increasingly recognized. Although its pathophysiology is not fully elucidated, associations with chronic inflammation, free radical release, Fas gene dysfunction, and tumor promotion in scar tissue have been suggested. Early identification of suspicious clinical changes and histopathological confirmation are essential, as SCC variants secondary to CBM tend to be aggressive. In addition, multidisciplinary care is required, as cases of this disease often show a limited or only initially favorable response despite prolonged antifungal treatment.^21^

This report represents the largest case series of this neglected disease with SCC development in a single hospital center worldwide, underscoring the need for increased resources, improved training of primary healthcare personnel, and appropriate treatment to prevent severe sequelae, including permanent disability and death.

## ORCID IDs

Edoardo Torres Guerrero: 0000-0003-2394-2730.

Héctor Proy Trujillo: 0000-0002-8023-2148.

María Elisa Vega Memije: 0000-0001-7985-118X.

Carlos Atoche Diéguez: 0000-0003-0811-1541.

## Research data availability

The entire dataset supporting the results of this study was published in this article.

## Financial support

None declared.

## Authors' contributions

Eduardo Rafael Calderón Quiroz: Approval of the final version of the manuscript; critical literature review; data collection, analysis, and interpretation; manuscript critical review; preparation and writing of the manuscript; statistical analysis; study conception and planning.

Edoardo Torres Guerrero: Approval of the final version of the manuscript; critical literature review; data collection, analysis, and interpretation; effective participation in research orientation; manuscript critical review; preparation and writing of the manuscript; study conception and planning.

Héctor Proy Trujillo: Data collection, analysis, and interpretation; effective participation in research orientation; intellectual participation in the propaedeutic and/or therapeutic management of the studied cases; manuscript critical review; statistical analysis; study conception and planning.

María Elisa Vega Memije: Data collection, analysis, and interpretation; effective participation in research orientation; intellectual participation in the propaedeutic and/or therapeutic management of the studied cases; statistical analysis; study conception and planning.

Carlos Atoche Diéguez: Data collection, analysis, and interpretation; effective participation in research orientation; intellectual participation in the propaedeutic and/or therapeutic management of the studied cases; manuscript critical review; statistical analysis; study conception and planning.

## Conflicts of interest

None declared.
